# Editorial: Advances in non-clinical and translational studies: cutting-edge study designs, special technologies, routine pitfalls, background findings and control data

**DOI:** 10.3389/fvets.2025.1743861

**Published:** 2026-01-12

**Authors:** Klaus Weber, A. Wallace Hayes, Dirk Schaudien, Ricardo de Miguel

**Affiliations:** 1 AnaPath Services GmbH, Liestal, Switzerland; 2 University of South Florida, Tampa, FL, United States; 3 Fraunhofer Institute for Toxicology and Experimental Medicine (FHG), Hannover, Germany

**Keywords:** laboratory animals, translational medicine, non-clinical studies, new alternative methodologies, enhanced toxicology interpretation, background findings

Non-clinical and translational research sits at a difficult crossroads as it must answer increasingly subtle questions about efficacy and safety while honoring 3R commitments and high animal welfare standards. As designs grow more complex and readouts more multidimensional, interpretation—not just measurement—can become the rate-limiting step. This Research Topic tackles that interpretive gap head-on. Across eight publications, it delivers tools, lexicons, and decision frameworks which render complex studies more intelligible, reproducible, and humane.

Starting with a pragmatic example, mechanistic imaging often fails, not for lack of sensitivity but for lack of simultaneity across tissue layers. Baker and colleagues solve that practical problem with a two-layer confocal method that uses a piezo actuator to bring distinct layers into the same focal plane, enabling real-time, dual-color Ca^2+^ imaging of interstitial cells of Cajal and smooth muscle cells during colonic motor activity (Baker et al.). Their work represents a key advancement over conventional sequential Z imaging while employing commercially available actuators and standard confocal hardware to ease the barrier to adoption.

Pathologists know that delayed fixation can hinder clean readouts. This is a relatively common occurrence in non-clinical studies, as animals sometimes die during a study. These decedents hold key information for assessing the potential toxicity of pharmaceuticals and pesticides. However, the animal's death triggers a cascade of physiological and biochemical alterations in tissues, posing challenges to histopathological evaluation. Atlases of histopathological lesions in the rat brain and kidney can aid pathologists in better interpreting histopathological findings observed under suboptimal fixation conditions due to delayed necropsies (Weber et al., de Miguel et al.). In general, microscopic changes vary among cell types and locations. In the brain, glial cells display consistent alterations across the brain and across the CNS, whereas neurons exhibit two distinct histological patterns with regional variations among neuroanatomical locations (Weber et al.). In the kidneys, distal convoluted tubules and the inner stripe of the outer medulla show earlier changes in contrast with other renal structures (de Miguel et al.). Exsanguination modestly delays and attenuates histopathological changes in the kidneys while showing no effect on the brain. Bagged vs. ventilated storage conditions have no clear effect on the onset or progression of post-mortem histopathological changes.

High-dimensional immunophenotyping is powerful but noisy. Differentiating when a shift in a cell subset implies adversity and when it is normal biological variation can be challenging due to large inter- and intra-species variability. In this sense, large historical control-value databases for different animal species can support data interpretation and decision-making (Johnson et al.). Moreover, technology in this area has undergone massive evolution, for example, spectral cytometry can currently support the use of more than 30 simultaneous markers. These techniques usually involve output of an enormous amount of data with challenging interpretation. Decision trees have been developed to guide the linking of immune phenotypes with functional assays and risk-informed decisions (Johnson et al.). This kind of guidance streamlines the decision-making process and helps to turn complex measurements into consistent conclusions.

In the complex field of osteoimmunology and hypoparathyroidism, animal models represent ideal systems to replicate what is observed in humans, but selecting the best model is not straightforward (Butylina et al.). Rodent models support genetic and surgical manipulation, yet large animals may be preferable for biomechanical and pharmacologic endpoints—except that accessory parathyroid glands complicate complete parathyroidectomy. Butylina et al. insightfully review this topic to address how PTH intersects with immune pathways and then frame a deliberate agenda for model selection in a disease where bone and immunity intertwine.

New approach methodologies (NAMs) have experienced vast development in recent years and are considered the future of many toxicology readouts. These techniques can enhance the non-clinical programs of multiple pharmaceuticals. Cardiovascular liabilities still drive expensive late attrition. Berridge and colleagues propose a multi-tiered, mechanistic, human-relevant screening paradigm positioned earlier in discovery, pairing human cell systems with *in vitro* to *in vivo* extrapolation (IVIVE) and explicit “failure modes” that map molecular perturbations to organ-level dysfunctions (Berridge et al.). This approach can help to speed up the acquisition of translatable results and reduce animal use, reserving them for targeted questions after mechanistic risks have been triaged with NAMs. This stepwise approach optimizes the interpretation of animal-specific findings and improves clinical biomarker strategies.

By contrast with other sectors, medical device biocompatibility has been slower to embrace alternatives in different research domains, but this is gradually changing (Kand′árová and Pôbiš). In fact, ISO 10993 23:2021 underscores prioritizing reconstructed human epidermis assays for skin irritation. Deep analysis of the root causes associated with the slower adoption of alternative methodologies, despite regulators' awareness, seems to pinpoint the partial lack of harmonization of protocols. In this sense, method standardization must catch up to achieve scientific and ethical gains (Kand′árová and Pôbiš).

PK/PD is a field in continuous development, boosted by modeling advances and increased computational power. Indeed, dose selection is a key step in the pipeline of therapeutic approaches. A good illustration is a study on tulathromycin using a neutropenic guinea pig model of *Haemophilus parasuis*, which demonstrated that the dose needed for a 2 log10 reduction was much lower when targeting AUC0−72 h/MIC than AUC0−168 h/MIC, arguing that the 72-h horizon may better reflect clinical goals (Guo et al.). The broader lesson here may be that PK/PD conclusions vary depending on the index and temporal window chosen, and these choices need to be rationally explained and reported transparently.

In conclusion, optimization of the readouts in non-clinical studies is ongoing with the aim of bolstering human translation. The immediate payoff is fewer false alarms, fewer missed real signals, and more informative studies. The long-term payoff is a culture that values clever measurements and the appropriate framework and stepwise approaches that make those measurements matter.

